# Changes in incidence of hospitalization for cardiovascular diseases during the COVID-19 pandemic in The Netherlands in 2020

**DOI:** 10.1038/s41598-023-39573-w

**Published:** 2023-08-08

**Authors:** Eva K. Kempers, Qingui Chen, Chantal Visser, Eric C. M. van Gorp, Frederikus A. Klok, Suzanne C. Cannegieter, Marieke J. H. A. Kruip

**Affiliations:** 1 Department of Hematology, Erasmus MC, Erasmus University Medical Center, Dr. Molewaterplein 40, 3015 GD Rotterdam, The Netherlands; 2 Department of Clinical Epidemiology, Leiden University Medical Center, Leiden, The Netherlands; 3 Department of Viroscience, Erasmus MC, Erasmus University Medical Center, Rotterdam, The Netherlands; 4 Department of Infectious Diseases, Erasmus MC, Erasmus University Medical Center, Rotterdam, The Netherlands; 5 Department of Medicine-Thrombosis and Hemostasis, Leiden University Medical Center, Leiden, The Netherlands

**Keywords:** Epidemiology, Cardiovascular diseases, Acute coronary syndromes, Arrhythmias, Heart failure, Vascular diseases, Viral infection, Public health, Epidemiology

## Abstract

This population-based cohort study aimed to describe changes in incidence of cardiovascular disease (CVD) hospital diagnoses during the COVID-19 pandemic in The Netherlands compared with the pre-pandemic period. We used Dutch nationwide statistics about hospitalizations to estimate incidence rate ratios (IRR) of hospital diagnoses of CVD during the first and second COVID-19 waves in The Netherlands in 2020 versus the same periods in 2019. Compared with 2019, the incidence rate of a hospital diagnosis of ischemic stroke (IRR 0.87; 95% CI 0.79–0.95), major bleeding (IRR 0.74; 95% CI 0.68–0.82), atrial fibrillation (IRR 0.73; 95% CI 0.65–0.82), myocardial infarction (IRR 0.78; 95% CI 0.72–0.84), and heart failure (IRR 0.74; 95% CI 0.65–0.85) declined during the first wave, but returned to pre-pandemic levels throughout 2020. However, the incidence rate of a hospital diagnosis of pulmonary embolism (PE) increased during both the first and second wave in 2020 compared with 2019 (IRR 1.30; 95% CI 1.15–1.48 and IRR 1.31; 95% CI 1.19–1.44, respectively). In conclusion, we observed substantial declines in incidences of CVD during the COVID-19 pandemic in The Netherlands in 2020, especially during the first wave, with an exception for an increase in incidence of PE. This study contributes to quantifying the collateral damage of the COVID-19 pandemic.

## Introduction

Excess mortality during the COVID-19 pandemic has been reported both at a global and national level^1,2^. The World Health Organization defines excess mortality as “*the mortality above what would be expected based on the non-crisis mortality rate in the population of interest*”^3^. The expected mortality is based on past trends and it represents the hypothetical scenario when the COVID-19 pandemic would not have occurred^1,2^. In The Netherlands, the cumulative excess deaths in 2020 and 2021 are estimated at a total of 30,000^2^. The total number of deaths in The Netherlands in this period amounted to 341,508^2^. During the first and second wave of the COVID-19 pandemic in The Netherlands, the excess mortality could be fully attributed to deaths due to COVID-19, whereas during the second half of 2021 only 70% could be explained by deaths due to COVID-19^2^. Causes of this excess mortality during the second half of 2021 remain to be determined, however, the impact of the COVID-19 pandemic on healthcare and public health, either direct or indirect, may be an important contributing factor.

Regarding the direct impact (i.e. because of SARS-CoV-2 infection), a high incidence of venous thromboembolism (VTE) has been reported in COVID-19 patients requiring hospitalization: 10% in the general ward and 28% in the intensive care unit (ICU) setting^4^. Moreover, a UK biobank study showed that the risk of myocardial infarction, stroke, heart failure, atrial fibrillation, VTE, and pericarditis was increased in individuals hospitalized with COVID-19 compared with matched uninfected controls^5^. These increased risks seemed not to be limited to hospitalized COVID-19 cases, as SARS-CoV-2 infection has also been associated with increased risk of various cerebrovascular and cardiovascular conditions among asymptomatic and non-hospitalized individuals^6,7^. However, others reported that among non-hospitalized infected individuals the increased risk was limited to incident VTE^5^.

The indirect impact of the pandemic on healthcare and public health might be substantial and could be both positive and negative. For example, there was delay or cancellation of routine care (e.g., non-urgent procedures, cancer screening), delay in seeking medical care, changes in lifestyle (including physical inactivity), less road traffic, decreased incidence of influenza and other common seasonal respiratory viruses, and potentially changed compliance to chronic medications during the COVID-19 pandemic^8–15^. The indirect effect of the pandemic on patients without COVID-19 has also been demonstrated by decreases in hospitalizations, accompanied with an increase in in-hospital mortality for a broad range of non-COVID-19 diseases during March to May and October to December 2020 in the United States^16^.

It is therefore relevant to have a comprehensive overview of the temporal distributions of various non-COVID-19 diseases during the pandemic, which may help to reveal potentially overlooked issues and better prepare for oncoming disease burden. Substantial declines in diagnosis of cerebrovascular events and some cardiovascular conditions (hypertension, type 2 diabetes, lipid disorders and atrial fibrillation) have already been reported in The Netherlands during the COVID-19 pandemic, but in primary care only^17^. The reported reduction in the number of first transient ischemic attacks (TIA) diagnoses was 37% and 29% for first stroke diagnoses, whereas the number of first diagnoses of cardiovascular events remained stable^17^.

However, few studies have been performed on the impact of the COVID-19 pandemic on the incidence of cardiovascular diseases in hospital settings^18–20^ and these studies did not include a broader range of cardiovascular diseases, including VTE. Therefore, this nationwide study aims to describe the changes in incidence of hospital diagnoses of arterial and venous thromboembolic diseases and other cardiovascular diseases in The Netherlands during the COVID-19 pandemic compared with the pre-pandemic period.

## Material and methods

### Data sources

This study used data on the complete population in The Netherlands provided by Statistics Netherlands, which is a Dutch governmental institution that gathers and links de-identified individual data from various nationwide data sources. For this study, we used data on household income of all Dutch inhabitants, personal characteristics, mortality, and diagnoses registered during hospitalizations in Dutch hospitals retrieved from discharge letters. More details about these data sources are provided in the supplementary information (data sources) and have been previously described^21^. The study complied with the Declaration of Helsinki, and received an ethical approval from the Scientific Committee of the Department of Clinical Epidemiology of the Leiden University Medical Center (No. A182) with a waiver of participant consent because of the use of pre-existing, de-identified data only.

### Study outcomes

The outcome event was a diagnosis of one of the following cardiovascular diseases registered during hospitalizations, i.e. both primary and non-primary diagnoses, in Dutch hospitals during the study period: ischemic stroke, TIA, other arterial thromboembolism, intracranial hemorrhage, major and clinically relevant non-major bleeding, atrial fibrillation, myocardial infarction, heart failure, and VTE [including pulmonary embolism (PE), deep vein thrombosis (DVT) and other types of VTE]. Major and clinically relevant non-major bleeding included intracranial hemorrhage, intraocular bleeding, bleeding causing anemia, pericardial bleeding, bleeding from esophageal varices, hemothorax, intra-auricular bleeding, bleeding from esophageal, gastric, duodenal, peptic or gastrojejunal ulcers, gastrointestinal bleeding, hemoperitoneum, intra-articular bleeding, hematuria, abnormal uterine and vaginal bleeding and hemorrhage from respiratory passages. Other arterial thromboembolism included embolism and thrombosis of the aorta, arteries of upper and lower extremities, iliac artery, and other arteries. The selected diagnoses were identified according to the International Classification of Diseases codes 10th Revision (ICD-10). The ICD-10 codes used to identify our study outcomes are displayed in Supplementary Table S1.

In addition, to describe the COVID-19 pandemic and to provide an overview of all hospitalizations in The Netherlands in 2020, the time distribution of COVID-19 diagnoses and any diagnosis registered within hospitalizations in Dutch hospitals in 2020 were determined.

### Study design and study populations

The study was divided into two parts. First, weekly incidence rates of a registered hospital diagnosis of the studied cardiovascular diseases (both primary and non-primary diagnoses) between 2015 and 2020 were determined (Supplementary Fig. S1). For this part, the study period was between January 1st 2015 and December 31st 2020. The study population included all Dutch inhabitants who had a record in the data set on household income and who survived at least until the start of each week. Participants were followed until the end of the week, death, or first occurrence of the study outcome, whichever occurred first.

Second, incidence rates of a registered hospital diagnosis of one of the study outcomes (both primary and non-primary diagnoses) were determined during the first and second wave of the COVID-19 pandemic in The Netherlands in 2020 (i.e., week 11–19 and week 36–52, respectively^22^) and the corresponding periods in 2019 (Supplementary Fig. S2). For this part, the study population included Dutch inhabitants who survived at least until the start of the first or second wave and Dutch inhabitants who survived until the start of the corresponding periods in 2019. The corresponding study populations were also followed until the end of the study period, death, or first occurrence of the study outcome, whichever occurred first.

### Data analysis

Weekly incidence rates of a registered hospital diagnosis of the studied cardiovascular diseases were calculated by dividing the number of events by observation time expressed in person-years. The calculated weekly incidence rates of the study outcomes between 2015 and 2019 were used to develop a model to estimate the expected weekly incidence rates of the study outcomes in 2020, in the hypothetical scenario that the COVID-19 pandemic had not occurred. Poisson regression was used to fit this model. In case of significant overdispersion, Quasi-Poisson regression was used. The following covariates were included in the model: week numbers (from 1 to 52), age groups (< 50, 50–59, 60–69, 70–79, ≥ 80 years), sex, immigration background (native Dutch, first generation of immigrants, second generation of immigrants), and household income level (categorized into low, 0–60% percentile, and high, 60–100% percentile). The natural logarithm of the observation time was used as an offset variable to account for the variation in duration of follow-up.

Incidence rate ratios (IRRs) of a registered hospital diagnosis of one of the study outcomes during the first and second wave of the COVID-19 pandemic in 2020 versus the same period in 2019 were determined. Poisson regression (or Quasi-Poisson regression in case of significant overdispersion) was used to estimate the IRRs of the investigated diseases between calendar years (i.e. 2020 vs 2019). Adjustment for age groups, sex, immigration background, household income level and comorbidities was performed. Comorbidities were determined by examining data on hospitalization within 5 years before the start of each study period for the following diseases: chronic obstructive pulmonary disease, other chronic lung disease (not including asthma), atrial fibrillation, heart failure, myocardial infarction, hypertension, rheumatic mitral stenosis/mechanical heart valves, other valvular heart diseases, liver diseases, chronic kidney diseases, anemia, diabetes, thyroid diseases, ischemic stroke, TIA, other arterial thromboembolism, Parkinson’s disease, Alzheimer’s disease, autoimmune diseases, system connective tissue disorders, VTE, major bleeding, and malignant tumors (Supplementary Table S2). The model was adjusted for comorbidities with a comorbidity index, which was calculated as the sum of the number of comorbidities and was categorized into 0, 1, 2, 3, and ≥ 4.

Data analyses were carried out in R, version 4.1.3, with the packages Stats 4.1.3, AER 1.2.9, ciTools 0.6.1, sandwich 3.0.1, lmtest 0.9.40, ggplot2 3.3.5 and dplyr 1.0.8^23–29^.

### Sensitivity analyses

A sensitivity analysis was performed by restricting to primary hospital admission diagnoses. In addition, since a low proportion of the diagnoses registered was imputed by Statistics Netherlands, the analyses were repeated without those imputed diagnoses as another sensitivity analysis.

## Results

### Population characteristics

Table 1 displays the personal characteristics of the study population at the start of the first wave of the COVID-19 pandemic in The Netherlands in 2020 and the corresponding period in 2019. Personal characteristics of the study population in the other periods, i.e. at the start of the second wave in 2020 and the corresponding period in 2019, are displayed in Supplementary Table S3. In total, 17,376,087 individuals were included in our analysis at the start of the first wave in 2020 (Table 1). The average age was 42.3 (± 23.4 SD) years and 50.3% was female. The majority of included individuals were native Dutch (75.7%) and 9.7% of individuals had a comorbidity index of ≥ 1.

**Table 1:** Personal characteristics of the study population at the start of the first wave in 2020 and the corresponding period in 2019 (week 11).

	First wave 2020	First wave 2019
	N = 17,376,087	N = 17,250,436
Age (years) mean (± SD)	42.3 (± 23.4)	42.2 (± 23.3)
Sex		
Males	8,632,681 (49.7%)	8,565,839 (49.7%)
Females	8,743,406 (50.3%)	8,684,597 (50.3%)
Immigration background		
Native Dutch	13,159,216 (75.7%)	13,167,676 (76.3%)
First generation immigrants	2,259,545 (13.0%)	2,159,005 (12.5%)
Second generation immigrants	1,957,326 (11.3%)	1,923,755 (11.2%)
Household income		
Low	7,726,956 (44.5%)	7,682,547 (44.5%)
High	9,649,131 (55.5%)	9,567,889 (55.5%)
Comorbidity index		
0	15,692,009 (90.3%)	15,601,084 (90.4%)
1	868,976 (5.0%)	865,144 (5.0%)
2	401,160 (2.3%)	390,452 (2.3%)
3	208,235 (1.2%)	199,851 (1.2%)
≥ 4	205,707 (1.2%)	193,905 (1.1%)

Individuals were classified as native Dutch when both parents were born in The Netherlands, as first generation immigrant when the individual was born in a foreign country and of whom at least one parent was also born in a foreign country, and as second generation immigrant when the individual was born in The Netherlands and of whom at least one parent was born in a foreign country. Household income level was categorized into low (0–60% percentile) and high (60–100% percentile). Comorbidities were determined by examining data on hospitalization within 5 years before the start of each study period. The comorbidity index was calculated as the sum of the number of comorbidities.

### COVID-19 pandemic in The Netherlands

**Figure 1:**
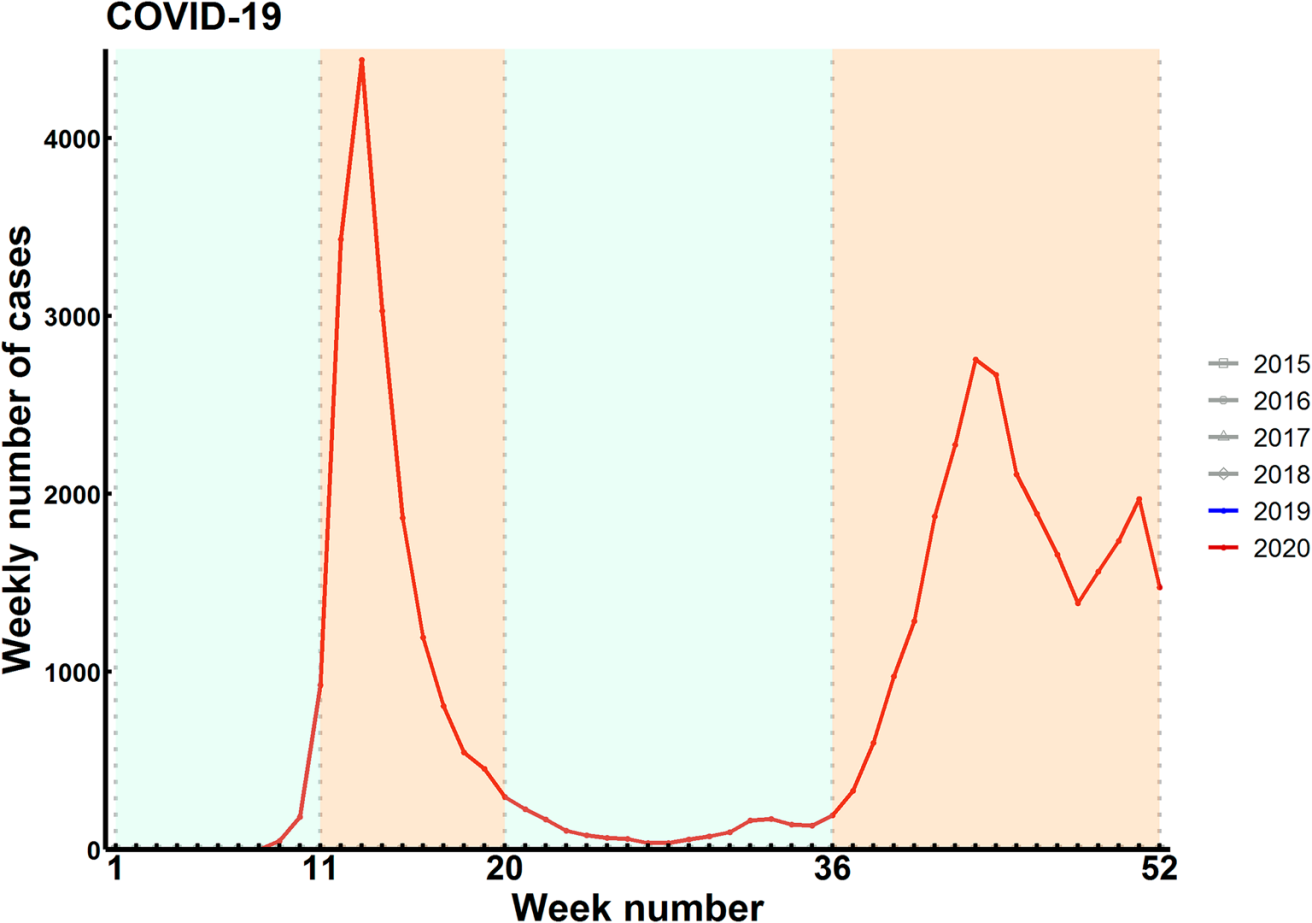
Weekly number of hospitalized COVID-19 cases in 2020. The orange shaded areas indicate the first and second wave of the COVID-19 pandemic in 2020 in The Netherlands.

The weekly numbers of hospitalized COVID-19 cases in 2020 in The Netherlands are displayed in Fig. 1. The incidence rate for any hospital admission was lower in 2020 than in previous years, especially during the first, but also during the second wave of the COVID-19 pandemic (Supplementary Fig. S3).

### Venous thromboembolism

**Figure 2:**
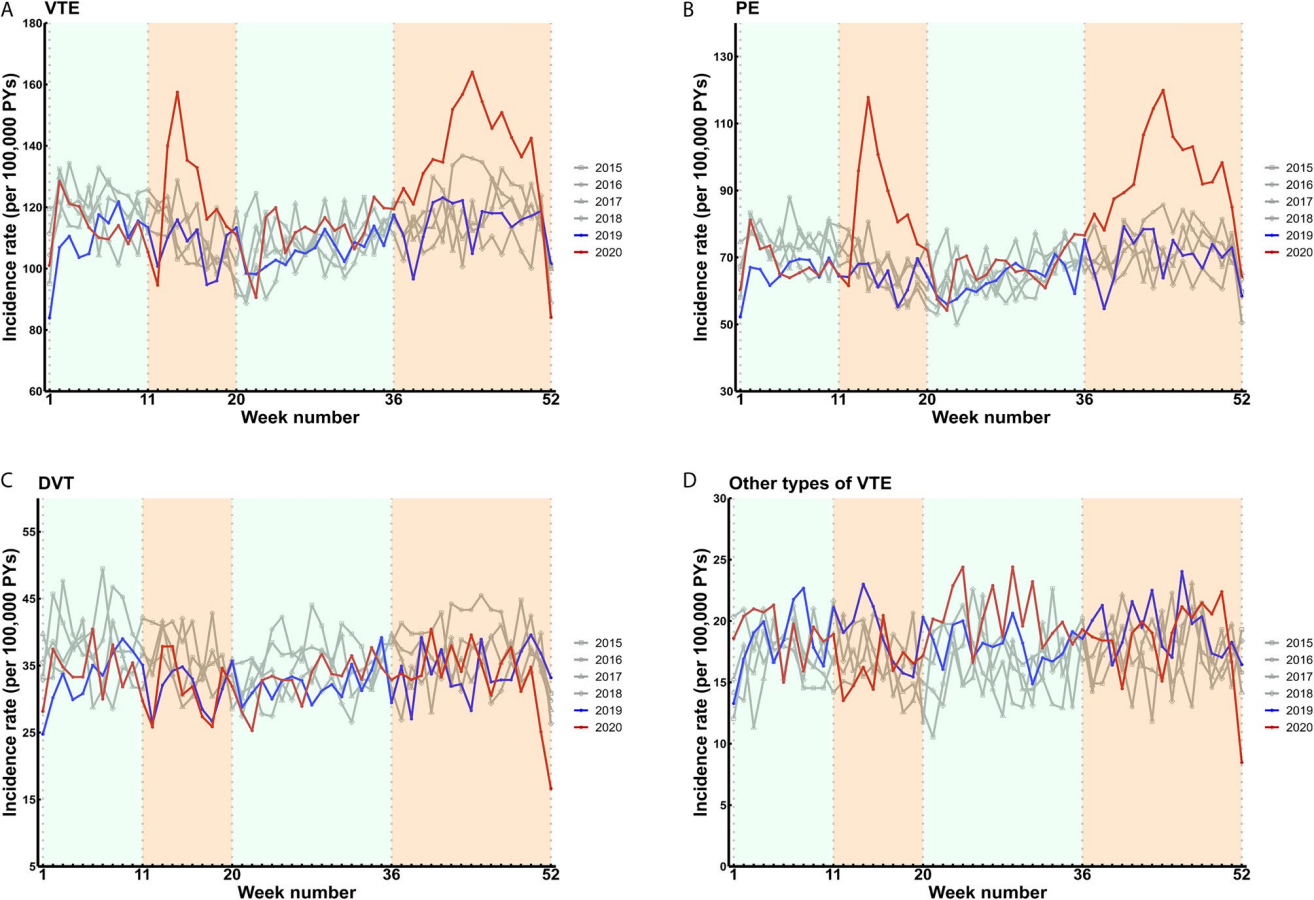
Weekly incidence rates of venous thromboembolism hospital diagnoses between 2015 and 2020. Incidence rates per 100,000 person-years (PYs). The orange shaded areas indicate the first and second wave of the COVID-19 pandemic in 2020 in The Netherlands. (**A**) Venous thromboembolism (VTE); (**B**) pulmonary embolism (PE); (**C**) deep vein thrombosis (DVT); (**D**) other types of venous thromboembolism (VTE).

The weekly incidence rates of a registered hospital diagnosis of VTE, including PE, DVT and other types of VTE, between 2015 and 2020 are displayed in Fig. 2. The incidence rate of a VTE hospital diagnosis increased during the first wave of the COVID-19 pandemic, declined again to pre-pandemic levels after the first wave, and showed a second peak during the second wave of the COVID-19 pandemic (Fig. 2a). Compared with 2019, the incidence rate of a hospital diagnosis of VTE in 2020 increased by 14% (IRR 1.14; 95% CI 1.00–1.29) during the first and by 17% (IRR 1.17; 95% CI 1.05–1.30) during the second wave (Fig. 5; Supplementary Table S4). This increase was predominantly driven by the concurrent rise in hospital diagnoses for PE in 2020 (Fig. 2b). More specifically, the incidence rate of a hospital diagnosis of PE in 2020 increased by 30% during both the first (IRR 1.30; 95% CI 1.15–1.48) and second wave (IRR 1.31; 95% CI 1.19–1.44) compared with 2019 (Fig. 5; Supplementary Table S4). In contrast, the incidence rates of a hospital diagnosis of DVT (Fig. 2c) and other types of VTE (Fig. 2d) were comparable to previous years throughout 2020.

### Arterial thromboembolism

**Figure 3:**
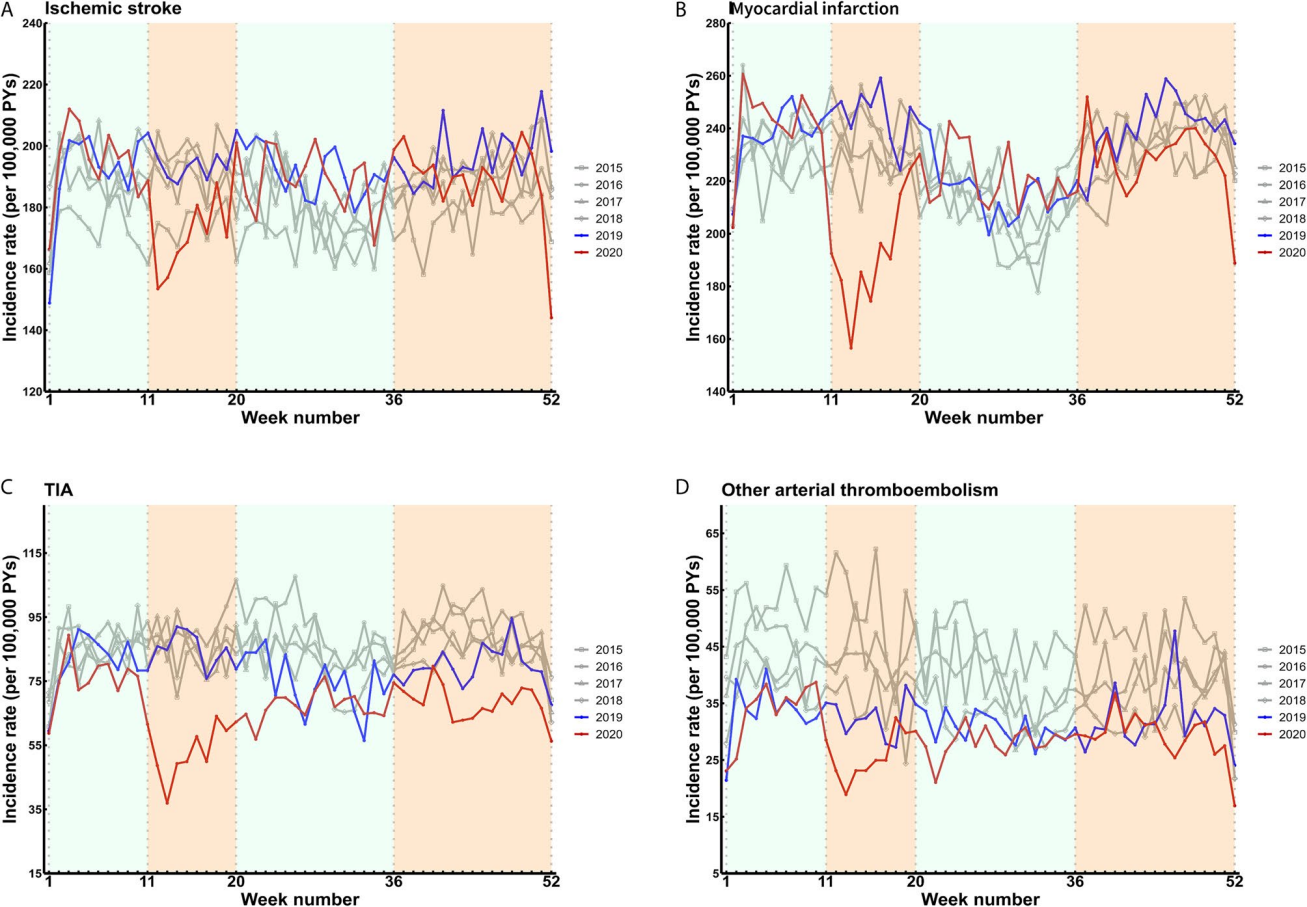
Weekly incidence rates of arterial thromboembolism hospital diagnoses between 2015 and 2020. Incidence rates per 100,000 person-years (PYs). The orange shaded areas indicate the first and second wave of the COVID-19 pandemic in 2020 in The Netherlands. (**A**) Ischemic stroke; (**B**) myocardial infarction; (**C**) transient ischemic attack (TIA); (**D**) other arterial thromboembolism.

The weekly incidence rates of a hospital diagnosis of ischemic stroke declined during the first wave of the COVID-19 pandemic and returned to pre-pandemic levels after the first wave throughout the rest of 2020, including the second wave (Fig. 3a). This decline during the first wave of the COVID-19 pandemic amounted to 13% (IRR 0.87; 95% CI 0.79–0.95) compared with 2019 (Fig. 5; Supplementary Table S4). A steep decline was observed in the weekly incidence rates of myocardial infarction hospital diagnoses from the start of the first wave in 2020, followed by an increased incidence rate after the first wave. During the second wave in 2020, the weekly incidence rates were lower compared with previous years, although less pronounced (Fig. 3b). Compared with 2019, this amounted to a 22% lower incidence rate of myocardial infarction during the first wave (IRR 0.78; 95% CI 0.72–0.84). However, the incidence rate of myocardial infarction returned to similar levels as 2019 during the second wave in 2020 (Fig. 5; Supplementary Table S4). The weekly incidence rates of a hospital diagnosis of TIA and other arterial thromboembolic events also declined during the first wave of the COVID-19 pandemic and remained lower compared with previous years throughout 2020 (Fig. 3c,d). Compared with the same time period in 2019, the incidence rates of TIA and other arterial thromboembolism were reduced by 38% (IRR 0.62; 95% CI 0.57–0.67) and 25% (IRR 0.75; 95% CI 0.66–0.85) during the first wave in 2020, respectively. Further declines were observed during the second wave for both TIA (IRR 0.85; 95% CI 0.79–0.91) and other arterial thromboembolism (IRR 0.90; 95% CI 0.80–1.00) compared with 2019 (Fig. 5; Supplementary Table S4).

### Other cardiovascular diseases

**Figure 4:**
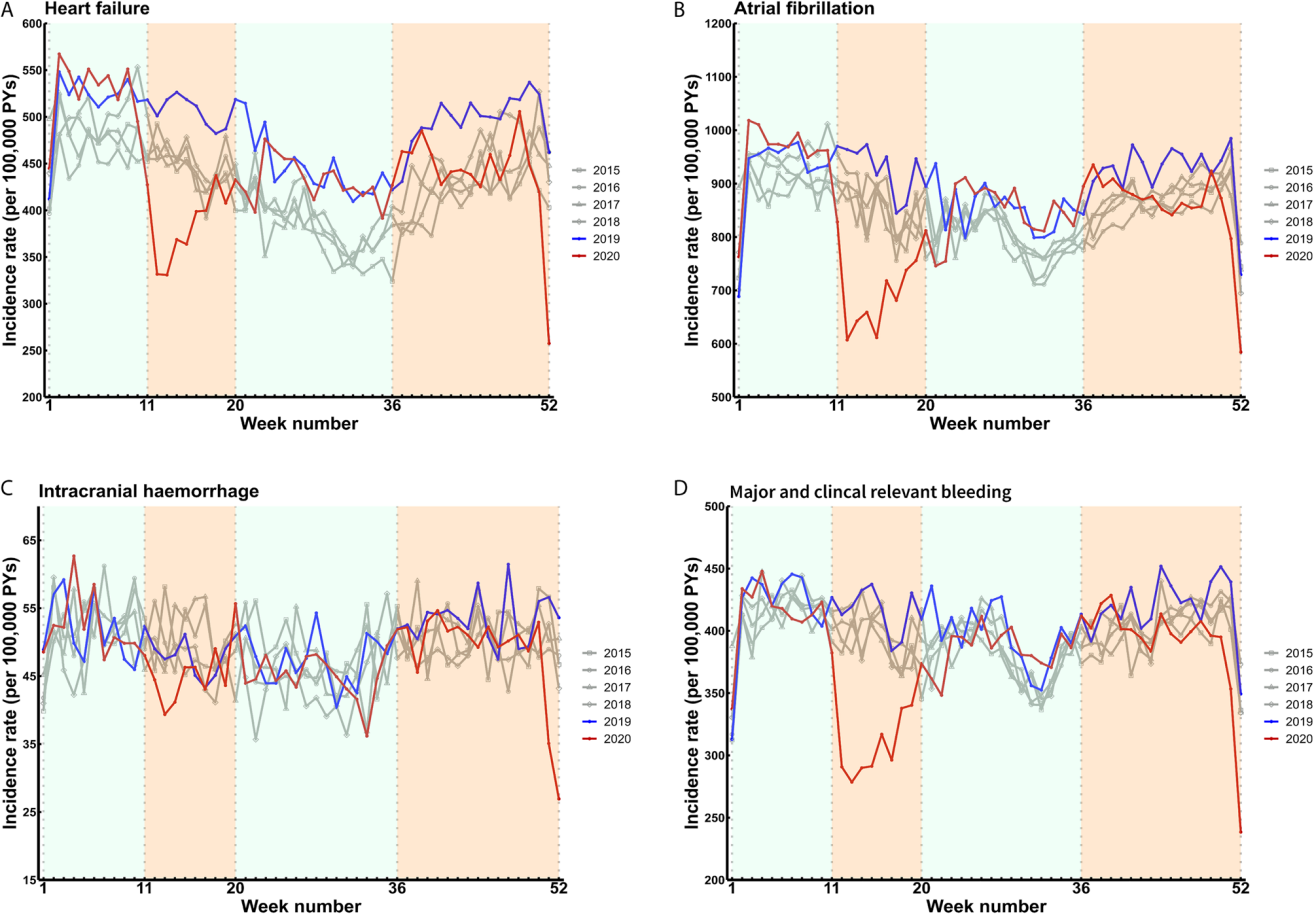
Weekly incidence rates of other cardiovascular disease hospital diagnoses between 2015 and 2020. Incidence rates per 100,000 person-years (PYs). The orange shaded areas indicate the first and second wave of the COVID-19 pandemic in 2020 in The Netherlands. (**A**) Heart failure; (**B**) atrial fibrillation; (**C**) intracranial hemorrhage; (**D**) major and clinically relevant non-major bleeding.

**Figure 5:**
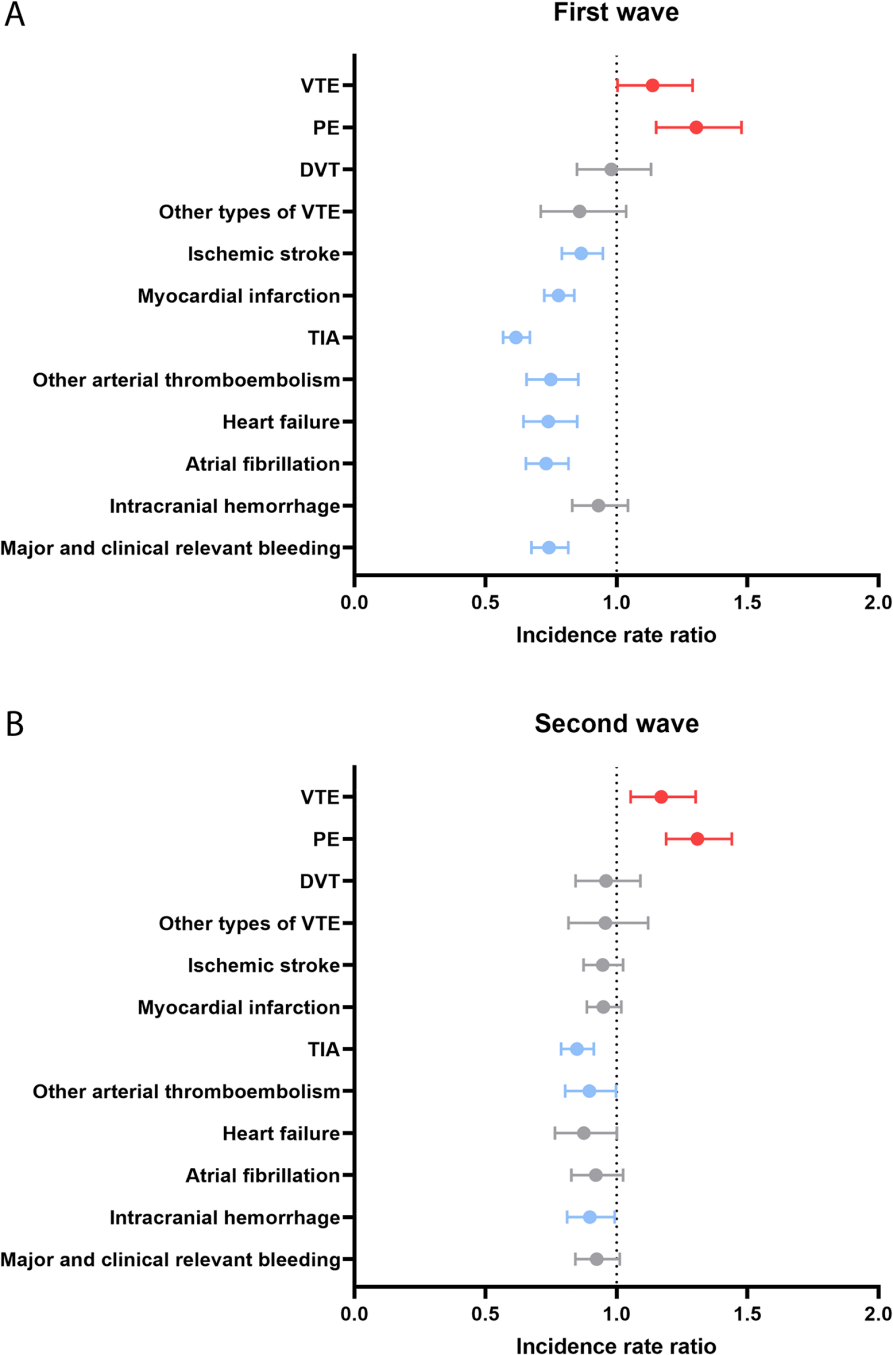
Incidence rate ratios of hospitalization during the first and second wave in 2020 versus 2019. Incidence rate ratios with 95% confidence intervals of hospitalization with a registered diagnosis of one of the study outcomes during both the first (**A**) and second (**B**) wave in 2020 separately versus the same time periods in 2019. *DVT* deep vein thrombosis, *PE* pulmonary embolism, *TIA* transient ischemic attack, *VTE* venous thromboembolism.

The weekly incidence rates of a registered hospital diagnosis of atrial fibrillation, heart failure, major and clinically relevant non-major bleeding and intracranial hemorrhage between 2015 and 2020 are displayed in Fig. 4. Declines in incidence rate during the first wave compared with the same period in 2019 were observed for major and clinically relevant non-major bleeding (IRR 0.74; 95% CI 0.68–0.82), atrial fibrillation (IRR 0.73; 95% CI 0.65–0.82), and heart failure (IRR 0.74; 95% CI 0.65–0.85). These incidence rates returned to pre-pandemic levels during the second wave in 2020 (Fig. 5; Supplementary Table S4). The incidence rate of a hospital diagnosis of intracranial hemorrhage during the first wave in 2020 was similar to 2019 (IRR 0.93; 95% CI 0.83–1.04), but declined by 10% during the second wave (IRR 0.90; 95% CI 0.81–0.99) (Fig. 5; Supplementary Table S4).

### Weekly incidence rates of hospitalization in 2020: observed vs expected

The observed versus expected weekly incidence rates of a registered hospital diagnosis of the studied cardiovascular diseases in 2020 showed roughly similar patterns as the actual incidence rates in 2020 versus 2015–19 (Supplementary Figs. S4–6).

### Sensitivity analyses

When restricting the analysis to the primary diagnosis registered with the hospital admission, the results were similar for most outcomes (Supplementary Table S5). However, the incidence rate of being admitted to a hospital for VTE during the first wave of the COVID-19 pandemic declined by 21% (IRR 0.79; 95% CI 0.70–0.89) compared with the same period in 2019. During the second wave of the COVID-19 pandemic the incidence rate of VTE was comparable to 2019 (IRR 0.97; 95% CI 0.87–1.08). Moreover, the incidence rate of hospital admission for PE during the first wave declined by 20% (IRR 0.80; 95% CI 0.71–0.90) and was comparable to 2019 during the second wave (IRR 0.97; 95% CI 0.87–1.08). In contrast, our initial analysis showed higher incidence rates of VTE, mainly driven by PE, during both the first and second wave in 2020 than in 2019. For a primary diagnosis of DVT, the incidence rate declined by 36% during the first wave compared with 2019 (IRR 0.64; 95% CI 0.52–0.79), while the incidence rate of DVT in our initial analysis was comparable to 2019.

When restricting the analysis to unimputed diagnoses only the results were similar for all outcomes.

## Discussion

In this large population-based cohort study in The Netherlands, we assessed the impact of the COVID-19 pandemic on the incidences of hospital diagnoses of arterial and venous thromboembolic diseases and other cardiovascular diseases during both the first and second wave of the COVID-19 pandemic in 2020. The incidence of a hospital diagnosis of VTE, predominantly driven by PE, increased during both the first and second wave of the COVID-19 pandemic compared with the same periods in 2019. In contrast, the incidences of ischemic stroke, myocardial infarction, TIA, other arterial thromboembolism, major and clinically relevant non-major bleeding, atrial fibrillation, and heart failure hospital diagnoses declined during the first wave in 2020 compared with 2019. Declines during the second wave were also observed for TIA, other arterial thromboembolism and intracranial hemorrhage. The incidences of the other cardiovascular diseases had returned to pre-pandemic levels after the first wave.

A possible explanation for the lower incidence of several cardiovascular disease hospital diagnoses we observed, especially during the first wave of the COVID-19 pandemic, could be that patients avoided or delayed seeking care. Healthcare avoidance by patients has been associated with the decline in consultation rate in both primary and specialist care during the COVID-19 pandemic^30^. A study performed within the general population in The Netherlands demonstrated that 20% of participants indicated that they had avoided seeking care during the first wave of the COVID-19 pandemic, despite experiencing symptoms for which they otherwise would have contacted their general practitioner or medical specialist^30^*.* Of whom 36% had experienced potentially urgent symptoms including chest pain, palpitations and limb weakness^30^. Most important determinants of healthcare avoidance were female sex, low self-appreciated health and high levels of anxiety and depression^30^. Other studies also reported reasons for healthcare avoidance including fear of contracting SARS-CoV-2 and not wanting to burden the health care system^14^.

Furthermore, general practitioners and elderly care physicians were also possibly more hesitant to refer patients to the emergency department because of the constrained healthcare resources during the pandemic and the perceived increased risk of infection. Cardiac-related attendances to emergency departments in England decreased during the first wave of the COVID-19 pandemic and reduction in rates of cardiovascular- and cerebrovascular-related referrals, diagnoses and treatments have been described in England, Italy and China^18^. Consequently, the clinical state of patients at admission was more severe during the COVID-19 pandemic compared with the pre-pandemic period^14^. These findings are not limited to cardiovascular diseases; a single-center retrospective study reported a 32% decrease in the number of trauma-related injuries present at the emergency department in The Netherlands^31^. Moreover, a Welsh study reported declines in incidence rates of several chronic conditions, such as mental health conditions and respiratory diseases, in 2020 and 2021 compared with expected rates^32^.

These potentially missed diagnoses of cardiovascular diseases can cause increased health complications and excess mortality, because acute treatment was not provided and appropriate secondary preventive care was not initiated^17,18^. Therefore, our findings may be important in understanding the excess mortality which was observed during the COVID-19 pandemic in The Netherlands^2^. Similar to The Netherlands, excess mortality was also observed in England from July to October 2021 and reductions in routine diabetes care delivery following the onset of the pandemic have been associated with this increase in non-COVID-19 related mortality in 2021^33^.

Another possible contributing factor to the lower incidence of cardiovascular diseases could be the competing risk of dying from COVID-19. This may have prevented at risk patients from developing the studied cardiovascular diseases. Additional studies are needed to determine whether the delayed and avoided care in 2020 has indeed led to increased incidence of complications and excess mortality in 2021.

The increased incidence of VTE hospital diagnoses during both the first and second wave of the COVID-19 pandemic may be attributed to the higher risk of VTE in hospitalized COVID-19 patients^4^. Previous studies demonstrated that the risk of VTE among hospitalized COVID-19 patients is higher than hospitalized patients with influenza, both before and during COVID-19 vaccine availability^34^. The increased VTE incidence in our study was predominantly driven by the rise in PE hospital diagnoses without a concurrent increase in DVT. In addition, differences between the results from our primary and sensitivity analysis suggest stronger positive associations between COVID-19 waves and non-primary hospital diagnoses of PE. This may provide further evidence that the increase in incidence of PE may be due to complications of COVID-19 in hospitalized patients. This finding may support the hypothesis that in COVID-19 patients, apart from conventional thromboembolism, pulmonary in-situ thrombosis might also occur^35^.

Another contributing factor to the increased incidences of VTE, in particular PE, observed in our study may be increased awareness and use of diagnostic imaging. Already early in the pandemic several studies reported a high incidence of thrombotic complications in hospitalized COVID-19 patients, especially in critically ill ICU patients^36^. Therefore, physicians may have been more inclined to send a COVID-19 patient in for diagnostic imaging. Over the course of the pandemic, the threshold for VTE suspicion and subsequent diagnostic testing in patients with COVID-19 was lowered^37^. Increased use of diagnostic imaging for PE in the second wave compared to the first wave of the COVID-19 pandemic has also been reported^38^. In addition, the number of diagnosed PEs limited to subsegmental arteries was higher in the second wave compared to the first wave, with an absolute increase of 14%^38^. Hence, as has been suggested previously, a part of the increased incidence of PE during the second wave may represent overdiagnosis^38^.

The increased incidence of VTE, predominantly driven by PE, during the COVID-19 pandemic may have important long-term consequences. A large proportion of PE survivors develop the post-pulmonary embolism syndrome 6 months after an acute PE^39^. This chronic complication negatively affects quality of life^40,41^. However, long-term complications after COVID-19 associated PE seem to be similar to non-COVID-19 associated PE^42^.

Our findings are consistent with previous studies on the impact of the COVID-19 pandemic on hospitalizations for cardiovascular diseases. A study on the global impact of the COVID-19 pandemic on stroke care reported an 11.5% decline in stroke hospitalizations during the pandemic compared to the pre-pandemic period^19^. In addition, hospitalization for acute coronary syndrome in England from February to May 2020 declined by 16% in comparison with 2019^20^.

Limitations of our study include the absence of data from non-hospitalized settings. Patients with TIA, atrial fibrillation and DVT for example are not necessarily admitted to a hospital and, therefore, less severe cases are possibly missed. This is especially the case during the COVID-19 pandemic because of constrained healthcare resources. The decline in incidence of TIA and atrial fibrillation hospital diagnoses may, therefore, overestimate the actual decline in incidence of these diagnoses during the first COVID-19 wave. However, declines in the number of first diagnoses of TIA, atrial fibrillation and stroke during the first COVID-19 wave have also been reported in the primary care setting^17^. The decline in incidences of most cardiovascular diseases may also be, at least partially, attributed to underregistration of diagnoses with the hospital admissions. Furthermore, data collected in the nationwide data sets is prone to misclassification, especially registration of the primary hospital admission diagnosis. It is therefore difficult to distinguish diagnoses present at admission from diagnoses that developed during hospitalization. This possible misclassification may have influenced the results of our sensitivity analysis, where we restricted to primary hospital admission diagnoses and observed lower incidence of VTE during the first wave of the pandemic.

Strengths of our study include our large study population, as we included data on the complete Dutch population, and that we assessed the impact of the COVID-19 pandemic on hospitalizations for a broad range of cardiovascular diseases, including venous and arterial thromboembolic diseases. This study, therefore, contributes to quantifying the collateral (health)damage of the COVID-19 pandemic and highlights that during future pandemics efforts should be made to prevent healthcare avoidance.

In conclusion, our population-based study describes relevant changes in hospitalization with cardiovascular diseases in The Netherlands during the COVID-19 pandemic compared with previous years. For most diseases, the incidence of hospitalization decreased, especially during the first wave of the COVID-19 pandemic. However, the incidence of a hospital diagnosis of PE increased during the COVID-19 pandemic compared with previous years. Additional studies are needed to determine the long-term impact of the COVID-19 pandemic because of delayed and avoided care and of COVID-19 associated health conditions, in particular COVID-19 associated PE.

### Supplementary Information

Supplementary Information.

## Data Availability

Results presented in the article were based on calculations by the authors using non-public microdata from Statistics Netherlands, but these data cannot be shared directly by the authors. Under certain conditions, these microdata are accessible for statistical and scientific research. For further information, contact: microdata@cbs.nl. All data analyzed during this study are included in this article.
