# Supplementary material for: Changes in incidence of hospitalization for cardiovascular diseases during the COVID-19 pandemic in The Netherlands in 2020
Source: Sci Rep. 2023 Aug 8;13:12832. doi: 10.1038/s41598-023-39573-w (PMC10409797; doi:10.1038/s41598-023-39573-w)
Supplement: Supplementary file 1 — Supplementary Information. [file 41598_2023_39573_MOESM1_ESM.pdf]

Changes in incidence of hospitalization for cardiovascular diseases during the COVID-19 pandemic in the Netherlands in 2020

Eva K. Kempers[1¶] (ORCID: 0000-0001-9458-8674), Qingui Chen[2¶] (ORCID: 0000-0001-9669-0007), Chantal Visser[1] (ORCID: 0000-0002-2025-1734), Eric C.M. van Gorp[3,4] (0000-0001-6415-6678), Frederikus A. Klok[5] (ORCID: 0000-0001-9961-0754), Suzanne C. Cannegieter[2,5] (ORCID: 0000-0003-4707-2303), Marieke J.H.A. Kruip[1*] (ORCID: 0000-0002-0265-4871)

[1]Department of Hematology, Erasmus MC, Erasmus University Medical Center, Rotterdam, The Netherlands.
[2]Department of Clinical Epidemiology, Leiden University Medical Center, Leiden, The Netherlands.
[3]Department of Viroscience, Erasmus MC, Erasmus University Medical Center Rotterdam The Netherlands.
[4]Department of Infectious Diseases, Erasmus MC, Erasmus University Medical Center Rotterdam The Netherlands.
[5]Department of Medicine - Thrombosis and Hemostasis, Leiden University Medical Center, Leiden, The Netherlands.

[*]Corresponding author
Marieke J. H. A. Kruip, MD PhD
Department of Hematology, Erasmus MC, Erasmus University Medical Center,
Dr. Molewaterplein 40, 3015 GD Rotterdam, The Netherlands.
Email: m.kruip@erasmusmc.nl

[¶]These authors contributed equally to this work.

SUPPLEMENTARY INFORMATION

Data sources

Data on household income were mainly collected from the Tax and Customs Administration and the student grant registrations of the Education Executive Agency (DUO). The population of the household income statistics includes all households in the Netherlands on January 1st of the reporting year.

Data on personal characteristics were collected from the Municipal Personal Records Database. Data on mortality were collected from the System of Social Statistical Files (“Sociaal-statistische Bestanden”, SSB) and covered all persons registered in the Municipal Personal Records Database in the Netherlands.

Data on diagnoses registered within hospitalizations were collected from the National Basic Register of Hospital Care of Dutch Hospital Data which included all general and academic Dutch hospitals and two short-stay categorical hospitals (i.e., a cancer clinic and an eye hospital).

Supplementary Table S1. ICD-10 codes used to identify study outcomes

Outcome	ICD-10 Code(s)
Ischemic stroke	I63, I64, H341
Transient ischemic attack	G45
Other arterial thromboembolism	I74
Intracranial hemorrhage	I60, I61, I62
Major bleeding and clinical relevant non-major bleeding	D62, D683, H313, H356, H431, H450, H922, I312, I60, I61, I62, I850, I9820, I983, J942, K2210, K2212, K2214, K2216, K228, K250, K252, K254, K256, K260, K262, K264, K266, K270, K272, K274, K276, K280, K282, K284, K286, K290, K3180, K5520, K625, K6380, K661, K920, K921, K922, M250, N02, N938, N939, N950, R04, R31, R58, S064, S065, S066
Atrial fibrillation	I48
Myocardial infarction	I21, I22
Heart failure	I50
Venous thromboembolism	I26, I801, I802, I803, I808, I809, I821, I822, I823, I828, I829, I81, I636, I676, G08, I820, G951, K550
Pulmonary embolism	I26
Deep vein thrombosis	I801, I802, I803, I808, I809, I821, I822, I823, I828, I829
Other types of venous thromboembolism	
Portal vein thrombosis	I81
Cerebral venous sinus thrombosis	I636, I676, G08
Budd-Chiari syndrome	I820
Vascular myelopathies	G951
Acute vascular disorders of intestine	K550
COVID-19	U071, U072

Supplementary Table S2. ICD-10 codes used to calculate the comorbidity index

Comorbidities for calculating number of comorbidities*	Count	ICD-10
Chronic obstructive pulmonary disease	1	J44
Other chronic lung diseases	1	J41, J42, J43, J47, J6, J7
Atrial fibrillation	1	I48
Heart failure	1	I50
Myocardial infraction (or history)	1	I21, I22, I23, I252
Hypertension	1	I10, I11, I12, I13, I15
Rheumatic mitral stenosis/Mechanical heart valves	1	I050, I052, I059, Z952
Other valvular heart diseases		I051, I058, I06, I07, I08, I34, I35, I36, I37, I38, I39
Liver diseases	1	B15, B16, B17, B18, B19, C22, K7, I85, I982, I983, Z944
Chronic kidney diseases	1	E102, E112, E122, E132, E142, I120, I13, N0, N1, N26, N27, Q60, Q61, T824, T861, Y602, Y612, Y622, Y841, Z49, Z940, Z992
Anaemia	1	D5, D60, D61, D62, D63, D64
Diabetes	1	E10, E11, E12, E13, E14
Thyroid diseases	1	E0
Ischemic stroke (or history)	1	I63, I64, H341, I693, I694
Transient ischemic attack		G45
Other arterial thromboembolism		I74
Parkinson's disease	1	G20
Alzheimer's disease		G30
Autoimmune diseases	1	D8
System connective tissue disorders		M3
Venous thromboembolism	1	I801, I802, I803, I808, I809, I821, I822, I823, I828, I829, I81, I820, K550, I636, I676, G08, G951, I26
Major bleeding	1	D62, D683, H313, H356, H431, H450, H922, I312, I60, I61, I62, I850, I9820, I983, J942, K2210, K2212, K2214, K2216, K228, K250, K252, K254, K256, K260, K262, K264, K266, K270, K272, K274, K276, K280, K282, K284, K286, K290, K3180, K5520, K625, K6380, K661, K920, K921, K922, M250, N02, N938, N939, N950, R04, R31, R58, S064, S065, S066
Malignant tumours	1	C

* by examining diagnoses registered within a hospitalization within five years before the index dates

Supplementary Table S3. Personal characteristics of the study population

	**First wave 2020**	**Second wave 2020**	**First wave 2019**	**Second wave 2019**
	N=17376087	N=17297089	N=17250436	N=17180402
**Age** (years) *mean (± SD)*	42.3 (±23.4)	42.6 (±23.3)	42.2 (±23.3)	42.5 (±23.2)
**Sex**				
Males	8632681 (49.7%)	8593294 (49.7%)	8565839 (49.7%)	8531620 (49.7%)
Females	8743406 (50.3%)	8703795 (50.3%)	8684597 (50.3%)	8648782 (50.3%)
**Immigration background**				
Native Dutch	13159216 (75.7%)	13091604 (75.7%)	13167676 (76.3%)	13107202 (76.3%)
First generation immigrants	2259545 (13.0%)	2252704 (13.0%)	2159005 (12.5%)	2153405 (12.5%)
Second generation immigrants	1957326 (11.3%)	1952781 (11.3%)	1923755 (11.2%)	1919795 (11.2%)
**Household income**				

Low	7726956 (44.5%)	7657495 (44.3%)	7682547 (44.5%)	7621462 (44.4%)
High	9649131 (55.5%)	9639594 (55.7%)	9567889 (55.5%)	9558940 (55.6%)
**Comorbidity index**				
0	15692009 (90.3%)	15621494 (90.3%)	15601084 (90.4%)	15514514 (90.3%)
1	868976 (5.0%)	857782 (5.0%)	865144 (5.0%)	866840 (5.0%)
2	401160 (2.3%)	401969 (2.3%)	390452 (2.3%)	395582 (2.3%)
3	208235 (1.2%)	208741 (1.2%)	199851 (1.2%)	203980 (1.2%)
≥4	205707 (1.2%)	207103 (1.2%)	193905 (1.1%)	199486 (1.2%)

Personal characteristics of the study population at the start of the first (week 11) and second (week 36) wave of the COVID-19 pandemic in the Netherlands and the corresponding periods in 2019. Individuals were classified as native Dutch when both parents were born in the Netherlands, as first generation immigrant when the individual was born in a foreign country and of whom at least one parent was also born in a foreign country, and as second generation immigrant when the individual was born in the Netherlands and of whom at least one parent was born in a foreign country. Household income level was categorized into low (0%-60% percentile) and high (60%-100% percentile). Comorbidities were determined by examining data on hospitalization within 5 years before the start of each study period. The comorbidity index was calculated as the sum of the number of comorbidities.

Supplementary Table S4. Incidence rate ratios of hospitalization with a diagnosis of one of the study outcomes in 2020 versus 2019

Outcome	First wave			Second wave		
	Crude IRR (95%CI)	Age and sex adjusted IRR (95%CI)	Fully adjusted IRR (95%CI)	Crude IRR (95%CI)	Age and sex adjusted IRR (95%CI)	Fully adjusted IRR (95%CI)
VTE	1.153 (0.651-2.041)	1.141 (0.938-1.387)	1.138 (1.004-1.290)	1.180 (0.676-2.060)	1.169 (0.983-1.391)	1.171 (1.054-1.302)
PE	1.319 (0.736-2.365)	1.305 (1.090-1.563)	1.304 (1.151-1.477)	1.319 (0.749-2.321)	1.306 (1.124-1.518)	1.309 (1.189-1.440)
DVT	0.992 (0.578-1.704)	0.983 (0.791-1.221)	0.980 (0.849-1.132)	0.966 (0.572-1.631)	0.958 (0.788-1.164)	0.960 (0.844-1.091)
Other types of VTE	0.873 (0.487-1.566)	0.865 (0.649-1.153)	0.859 (0.711-1.037)	0.965 (0.546-1.706)	0.957 (0.733-1.249)	0.957 (0.817-1.121)
Ischemic stroke	0.882 (0.448-1.735)	0.868 (0.748-1.006)	0.865 (0.791-0.948)	0.959 (0.489-1.881)	0.946 (0.823-1.086)	0.947 (0.874-1.025)
Myocardial infarction	0.792 (0.419-1.499)	0.782 (0.675-0.905)	0.779 (0.724-0.839)	0.961 (0.512-1.804)	0.950 (0.834-1.083)	0.950 (0.887-1.018)
TIA	0.626 (0.321-1.220)	0.616 (0.544-0.697)	0.616 (0.567-0.669)	0.859 (0.441-1.671)	0.847 (0.759-0.946)	0.849 (0.789-0.913)

Other arterial thromboembolism	0.764 (0.392-1.489)	0.753 (0.552-1.028)	0.749 (0.657-0.854)	0.905 (0.474-1.728)	0.894 (0.671-1.192)	0.896 (0.804-0.998)
Atrial fibrillation	0.750 (0.370-1.520)	0.736 (0.531-1.021)	0.731 (0.654-0.817)	0.934 (0.466-1.871)	0.920 (0.677-1.250)	0.921 (0.828-1.025)
Heart failure	0.765 (0.360-1.626)	0.751 (0.494-1.141)	0.740 (0.645-0.850)	0.892 (0.424-1.875)	0.878 (0.588-1.310)	0.875 (0.765-1.001)
Intracranial haemorrhage	0.947 (0.499-1.799)	0.934 (0.788-1.107)	0.931 (0.831-1.044)	0.908 (0.485-1.699)	0.897 (0.769-1.046)	0.898 (0.812-0.993)
Major and clinical relevant bleeding	0.755 (0.430-1.327)	0.746 (0.594-0.938)	0.742 (0.675-0.816)	0.932 (0.542-1.604)	0.923 (0.750-1.136)	0.924 (0.843-1.012)

Incidence rate ratios were computed compared to the same period in 2019. The fully adjusted model was adjusted for age groups, sex, immigration background, household income, and comorbidity index. DVT = deep vein thrombosis; IRR = incidence rate ratio; PE = pulmonary embolism; TIA = transient ischemic attack; VTE = venous thromboembolism.

Supplementary Table S5. Sensitivity analysis restricting the analysis to the primary diagnosis made within a hospitalization

Outcome	First wave			Second wave		
	Crude IRR (95%CI)	Age and sex adjusted IRR (95%CI)	Fully adjusted IRR (95%CI)	Crude IRR (95%CI)	Age and sex adjusted IRR (95%CI)	Fully adjusted IRR (95%CI)
VTE	0.796 (0.470-1.350)	0.788 (0.666-0.933)	0.789 (0.702-0.886)	0.974 (0.580-1.634)	0.965 (0.836-1.114)	0.969 (0.869-1.079)
PE	0.806 (0.466-1.396)	0.798 (0.673-0.946)	0.799 (0.707-0.903)	0.975 (0.568-1.673)	0.966 (0.845-1.105)	0.970 (0.870-1.080)
DVT	0.647 (0.403-1.037)	0.642 (0.499-0.827)	0.642 (0.524-0.785)	0.892 (0.578-1.377)	0.887 (0.716-1.100)	0.890 (0.751-1.055)
Other types of VTE	0.916 (0.527-1.591)	0.907 (0.740-1.112)	0.902 (0.736-1.106)	1.032 (0.609-1.747)	1.023 (0.781-1.341)	1.025 (0.848-1.240)
Ischemic stroke	0.882 (0.448-1.739)	0.868 (0.754-0.999)	0.866 (0.794-0.945)	0.957 (0.487-1.882)	0.944 (0.827-1.078)	0.945 (0.874-1.022)
Myocardial infarction	0.788 (0.417-1.490)	0.778 (0.682-0.888)	0.776 (0.721-0.836)	0.959 (0.512-1.796)	0.948 (0.841-1.069)	0.948 (0.885-1.016)
TIA	0.619 (0.317-1.207)	0.609 (0.543-0.684)	0.609 (0.562-0.661)	0.858 (0.441-1.669)	0.847 (0.764-0.940)	0.849 (0.790-0.912)
Other arterial thromboembolism	0.645 (0.329-1.266)	0.636 (0.459-0.880)	0.633 (0.557-0.718)	0.885 (0.465-1.686)	0.874 (0.654-1.170)	0.877 (0.782-0.984)
Atrial fibrillation	0.620 (0.318-1.208)	0.611 (0.447-0.836)	0.609 (0.544-0.682)	0.929 (0.482-1.790)	0.918 (0.687-1.225)	0.921 (0.826-1.027)

Heart failure	0.703 (0.328-1.506)	0.689 (0.449-1.058)	0.680 (0.590-0.783)	0.889 (0.418-1.890)	0.875 (0.582-1.316)	0.873 (0.755-1.010)
Intracranial hemorrhage	0.945 (0.498-1.795)	0.931 (0.796-1.090)	0.929 (0.832-1.038)	0.916 (0.488-1.721)	0.905 (0.781-1.050)	0.906 (0.824-0.996)
Major and clinical relevant bleeding	0.686 (0.396-1.187)	0.678 (0.553-0.831)	0.675 (0.619-0.736)	0.897 (0.532-1.512)	0.888 (0.739-1.068)	0.889 (0.819-0.964)

Sensitivity analysis restricting the analysis to the primary diagnosis made within a hospitalization. Incidence rate ratios were computed compared to the same period in 2019. The fully adjusted model was adjusted for age groups, sex, immigration background, household income, and comorbidity index. DVT = deep vein thrombosis; IRR = incidence rate ratio; PE = pulmonary embolism; TIA = transient ischemic attack; VTE = venous thromboembolism.

Supplementary Figure S1. Illustration of part I of the study

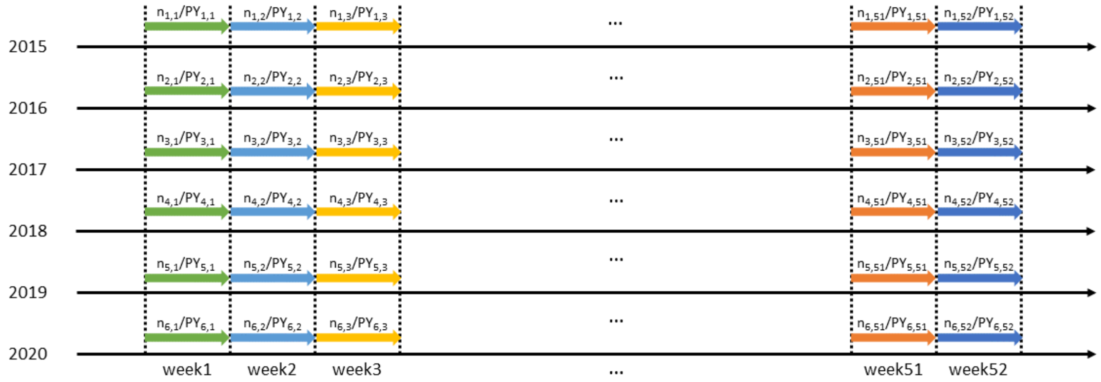

Supplementary Figure S2. Illustration of part II of the study

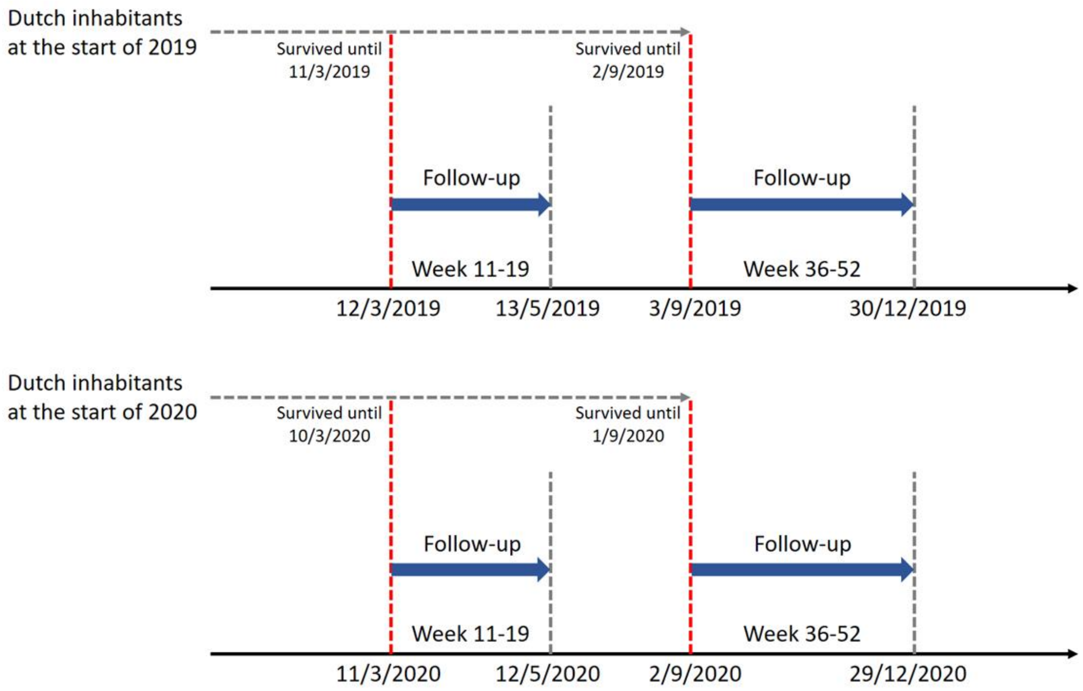

Supplementary Figure S3. Weekly incidence rates of any hospital admission between 2015 and 2020

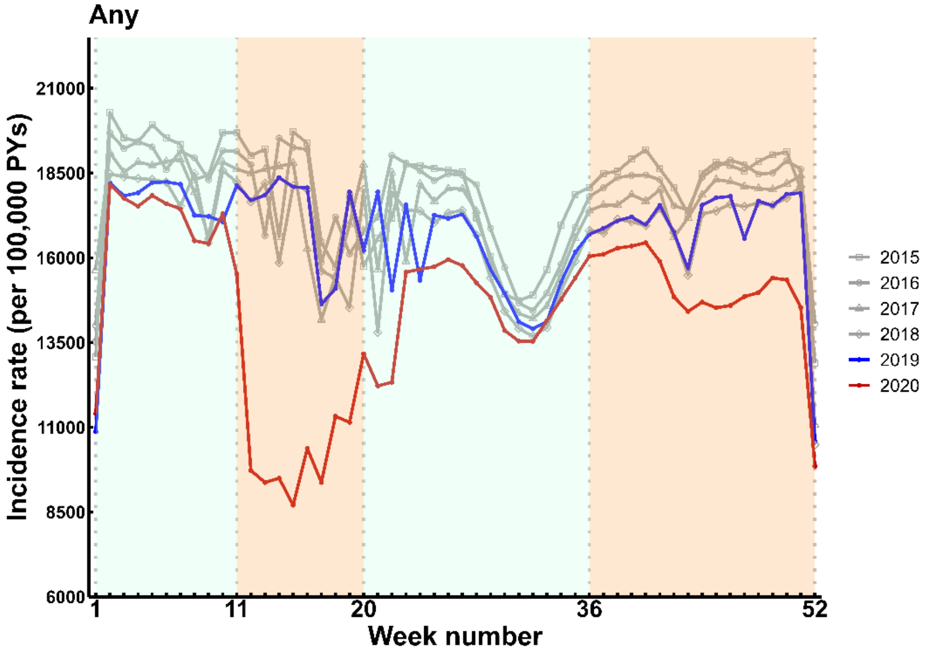

Incidence rates per 100,000 person-years (PYs). The orange shaded areas indicate the first and second wave of the COVID-19 pandemic in 2020 in the Netherlands.

Supplementary Figure S4. Observed versus expected weekly incidence rates of hospitalization with venous thromboembolism in 2020

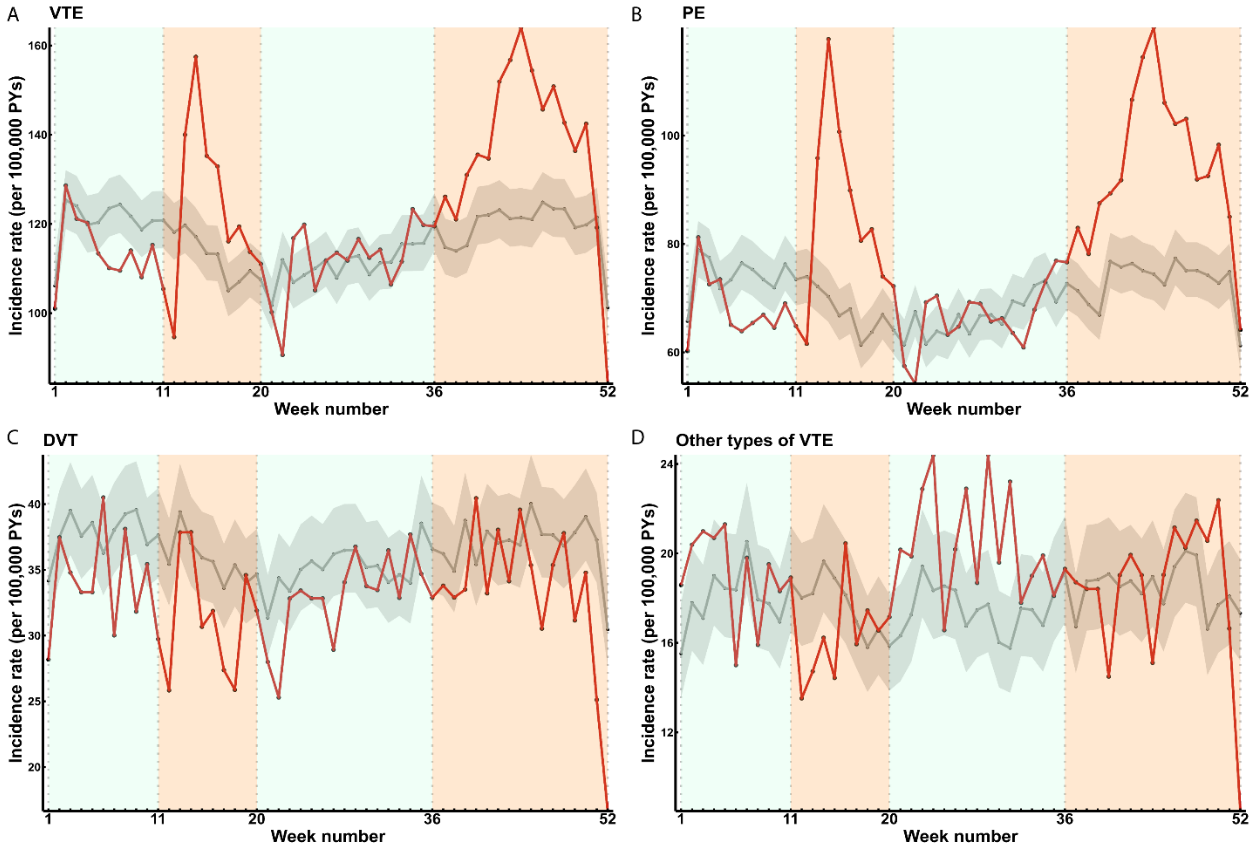

Incidence rates per 100,000 person-years (PYs). The orange shaded areas indicate the first and second wave of the COVID-19 pandemic in 2020 in the Netherlands. DVT = deep vein thrombosis; PE = pulmonary embolism; VTE = venous thromboembolism.

Supplementary Figure S5. Observed versus expected weekly incidence rates of hospitalization with arterial thromboembolism in 2020

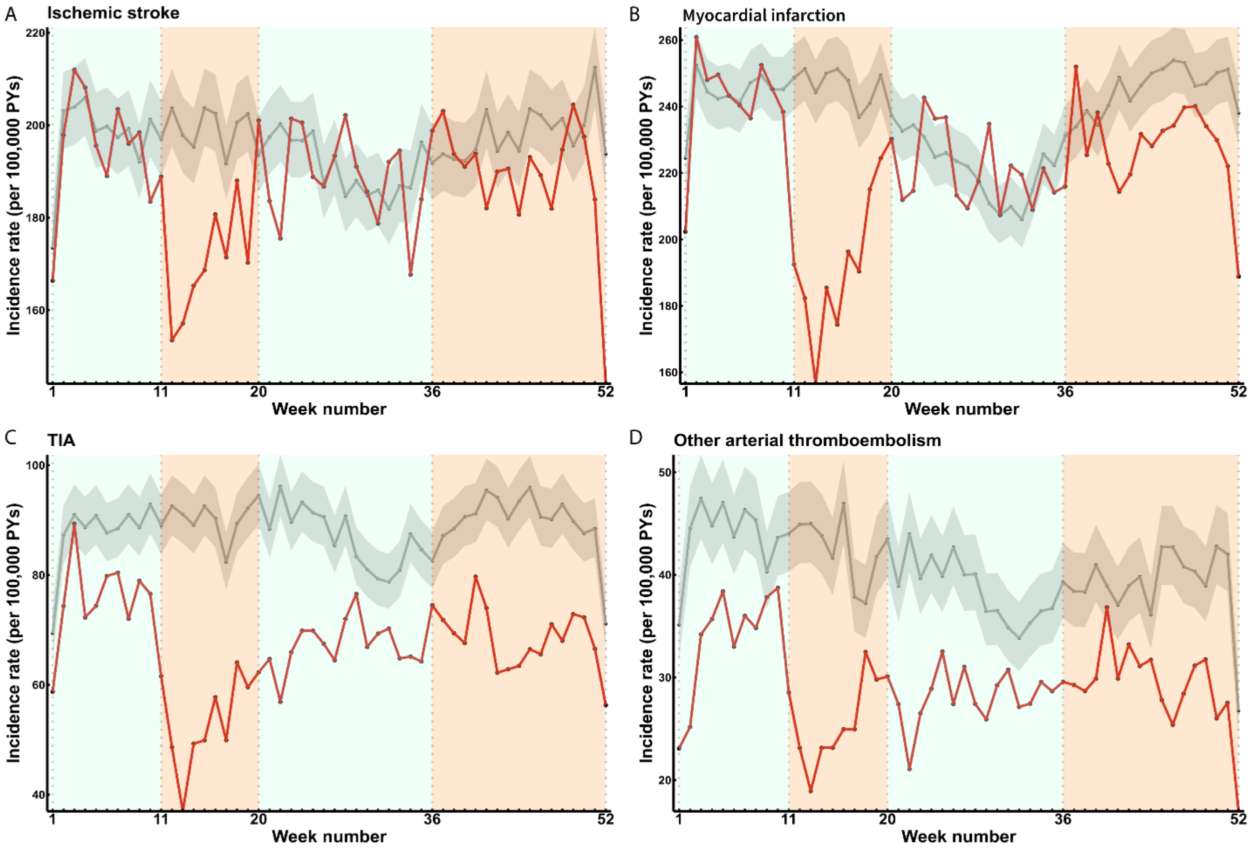

Incidence rates per 100,000 person-years (PYs). The orange shaded areas indicate the first and second wave of the COVID-19 pandemic in 2020 in the Netherlands. TIA = transient ischemic attack.

Supplementary Figure S6. Observed versus expected weekly incidence rates of hospitalization with other cardiovascular disease in 2020

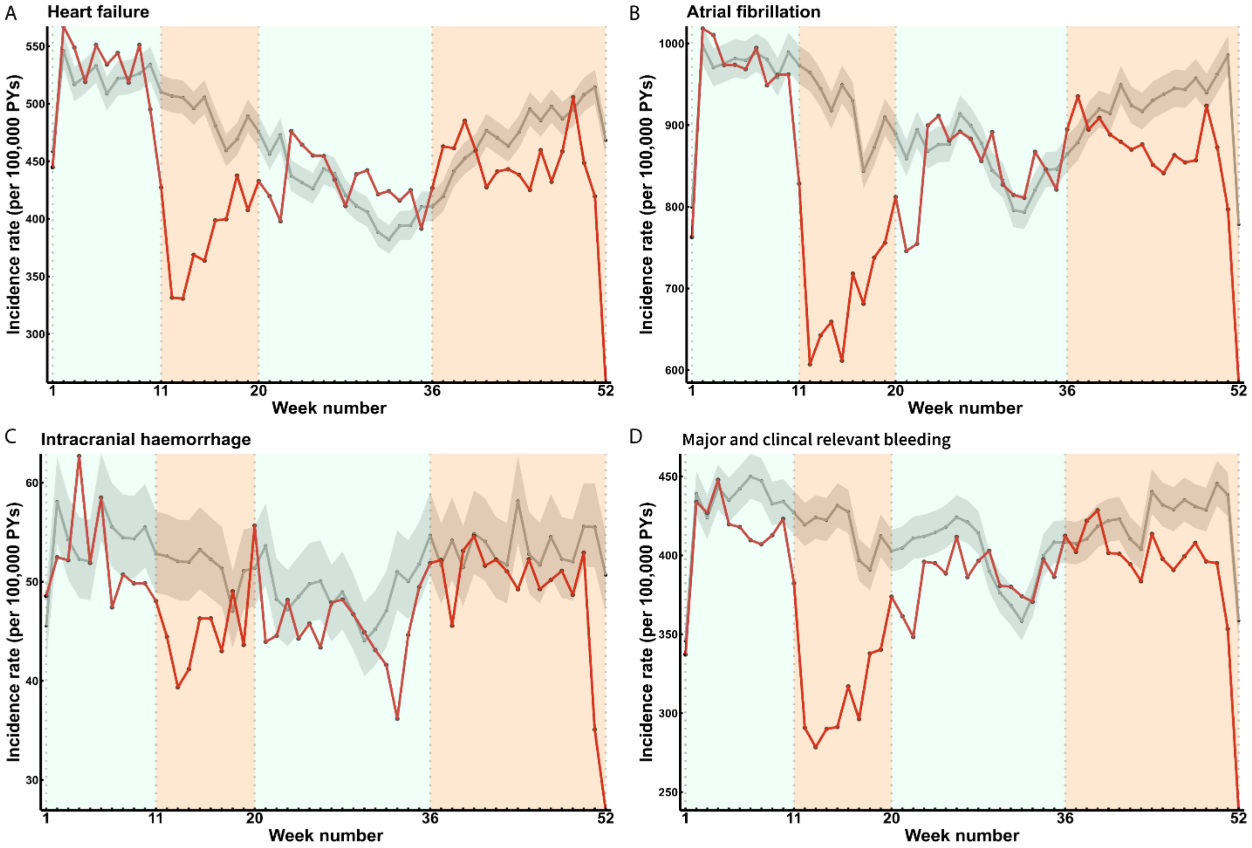

Incidence rates per 100,000 person-years (PYs). The orange shaded areas indicate the first and second wave of the COVID-19 pandemic in 2020 in the Netherlands.

Dutch COVID & Thrombosis Coalition consortium members

Amphia Hospital

Dr. M. J. J. H Grootenboers, pulmonologist

Dr. C van Guldener, internist

Dr. M. Kant, pulmonologist

Amsterdam University Medical Center

- Location AMC:

Prof. Dr. D. van de Beek, neurologist

Dr. M. C. Brouwer, neurologist

Drs S. de Bruin, phd-candidate

Dr. M. Coppens, internist vascular medicine

Drs. N. van Es, phd candidate

Drs. T. F. van Haaps, phd-candidate Department of Vascular Medicine

Prof. dr. N. P. Juffermans, intensivist

Dr. M. C. A. Muller, intensivist

Prof. Dr. A. P. J. Vlaar, intensivist

- Location VUMC:

Prof. dr. C. M. P. M. Hertogh, nursing home specialist

Prof. Dr. L. M. A. Heunks, professor intensive care

Drs. J. G. Hugtenburg, pharmacologist

Dr. J. van Kooten, nursing home specialist

Dr. E. J. Nossent, pulmonologist

Prof. Dr. Y. Smulders, internist

Dr. P. R. Tuinman, intensivist

Dr. A. Vonk Noordegraaf, pulmonologist

Argos Zorggroep

Drs. A. Lansbergen, physiotherapist

Deventer Hospital

Dr. J. Faber, coordinator of the research organisation

Dr. G. Hajer, internist vascular medicine

Dr. A. Stemerdink, intensivist

Erasmus Medical Center

Drs. J. van den Akker, intensivist

Dr. R. Bierings, cellular biologist, Department Haematology Dr. H. Endeman, intensivist Dr. M. Goeijenbier, internal medicine, Department Viroscience Prof. dr. D. A. M. P. J. Gommers, intensivist

Prof. dr. E. C. M. van Gorp, infectious diseases specialist, Department Viroscience Dr. N. G. M. Hunfeld, hospital pharmacist Prof. dr. M.P.G. Koopmans, internist, Department Viroscience

E. K. Kempers, phd-candidate, Department Haematology

Dr. M. J. H. A. Kruip, haematologist Prof. dr. T. Kuiken, professor of comparative pathology, Department Viroscience Drs. T. Langerak, phd-candidate, Department Viroscience

Dr. M. N. Lauw, haematologist, Department Haematology

Prof. Dr. Leebeek, professor of Haemostasis and Thrombosis

Prof. dr. M. P. M. de Maat, head of Biochemistry of Haemostasis and Thrombosis Drs. D. Noack, phd-candidate i.o. Department Viroscience

Drs. M.S. Paats, pulmonologist Drs. M.P. Raadsen, phd-candidate, Department Viroscience Dr. B. Rockx, assistant professors, Department Viroscience

Dr. C. Rokx, infectious diseases specialist

Dr. C. A. M. Schurink, infectious diseases specialist Drs. K. Tong-Minh, phd-candidate, Department Viroscience

Dr. L. van den Toorn, pulmonologist

Dr. C. A. den Uil, cardiologist-Intensivist

Drs. C. Visser, phd-candidate, Department Haematology

Farmadam

Drs. F. Boutkourt, phd-candidate

Drs. T. Roest, pharmacist

Flevoziekenhuis

Dr. R. A. Douma, infectious diseases specialist

Drs. L. R. de Haan, phd-candidate

Dr. M. ten Wolde, internist vascular medicine

Hospital Gelderse Vallei

Dr. R. H. H. Bemelmans, internist

Dr. B. Festen, intensivist

Ikazia Hospital

Dr. S. Stads, intensivist

Jeroen Bosch Hospital

Dr. C. P. C. de Jager, intensivist

Dr. K.S. Simons, intensivist

Leiden University Medical Center

Drs. M.L. Antoni, cardiologist, Department of Cardiology

Dr. M. H. Bos, biochemicus, associate professor, Department of Medicine - Thrombosis and Hemostasis

Drs. J. L. I. Burggraaf, phd-candidate, Department of Clinical Epidemiology

Prof. S. C. Cannegieter, clinical epidemiologist, Department of Medicine - Thrombosis and Hemostasis and Department of Clinical Epidemiology

Prof. dr. H. C. J. Eikenboom, haematologist/internist vascular medicine, Department of Medicine - Thrombosis and Hemostasis

Dr. P. L. den Exter, vascular medicine specialist, Department of Medicine - Thrombosis and Hemostasis

Dr. J. J. M. Geelhoed, pulmonologist, Department of Pulmonology

Prof. dr. M. V. Huisman, internist vascular medicine, Department of Medicine - Thrombosis and Hemostasis

Drs. C. M. M. de Jong, phd-candidate, Department of Medicine - Thrombosis and Hemostasis

Prof. E. de Jonge, internist-intensivist, Department of Intensive Care Medicine

Drs. F. H. J. Kaptein, phd-candidate, Department of Medicine - Thrombosis and Hemostasis

Dr. F. A. Klok, internist vascular medicine, Department of Medicine - Thrombosis and Hemostasis

Dr. L. J. M. Kroft, radioloog, Department of Radiology

Dr. W. M. Lijfering, Clinical epidemiologist, Department of Medicine - Thrombosis and Hemostasis

Drs. L. Nab, phd-candidate, Department of Clinical Epidemiology

Dr. M. K. Ninaber, pulmonologist, Department of Pulmonology

Prof. dr. H. Putter, statistician, Department of Biomedical Data Sciences

Drs. S. R. S. Ramai, pulmonologist, Department of Pulmonology

Dr. A. M. da Rocha Rondon, postdoctoral researcher, Department of Medicine - Thrombosis and Hemostasis

Dr. A. H. E. Roukens, infectious diseases specialist, Department of Infectious Diseases

Drs. M. A. M. Stals, phd-candidate, Department of Medicine - Thrombosis and Hemostasis

Prof. dr. H .H. Versteeg, cellular biologist, Department of Medicine - Thrombosis and Hemostasis

Dr. H. W. Vliegen, cardiologist, Department of Cardiology

Dr. B. J. M. van Vlijmen, cellular biologist, associate professor, Department of Medicine - Thrombosis and Hemostasis

Maastricht University Medical Center

Drs. T. van de Berg, phd-candidate

Drs. R. Bruggemann, phd-candidate

Dr. B. C. T. van Bussel, internist-intensivist

Prof. dr. H. ten Cate, internist

Dr. A. J. ten Cate-Hoek, clinicial epidemiologist and medical director of thrombosis service Maastricht

Prof. dr. T. M. Hackeng, biochemist

Dr. ir. Y. Henskens, clinical chemist

Drs. A. Hulshof, phd-candidate

Drs. M. Mulder, phd-candidate

Drs. R. H. Olie, internist vascular medicine

Prof. dr. L. Schurgers, biochemist

Dr. B. Spaetgens, internist subspecialised in geriatrics

Dr. H. Spronk, biochemist

Dr. K. Winckers, internist vascular medicine

Maxima Medical Center

Dr. L. Nieuwenhuizen, haematologist

Medical Center Leeuwaarden

Drs. B. Franken, haematologist

Dr. I. M. Schrover, internist vascular medicine

Drs. E. G. M. de Waal, haematologist

Medical Center Twente

Dr. A. Beishuizen, intensivist

Dr. A. Cornet, intensivist

Dr. J. Krabbe, clinical biochemist

Noordwestziekenhuisgroep

Dr. W. G. Boersma, pulmonologist

Drs. L. M. Hessels, phd-candidate

OLVG Hospital

Prof. dr. N. P. Juffermans, intensivist

Radboud University Medical Center

Dr. B. van den Borst, pulmonologist

Prof. dr. K. Kramers, professor medical safety Dr. J. Leentjens, internist vascular medicine Dr. Q. de Mast, infectious diseases specialist

Prof. dr. S. Middeldorp, internist vascular medicine

Reinier de Graaf Gasthuis

Dr. R. E. Brouwer, haematologist

Dr. J. L. J. Ellerbroek, infectious diseases specialist

Drs. J. Tijmensen, haematologist

Rijnstate Hospital

Dr. M. M. C. Hovens, internist vascular medicine

Dr. E. A. N. Oostdijk, intensivist

Drs. B. D. Westerhof, anaesthesiologist-intensivist

Rode Kruis Hospital

Dr. L. M. Faber, haematologist

Dr. F. S. Kleijwegt, haematologist

Sanquin Research, Amsterdam

Dr. M. van den Biggelaar, head of Laboratory of Proteomics, Department of Molecular and Cellular Hemostasis Prof. Dr. J. C. M. Meijers, biochemist (and Amsterdam University Medical Center) Prof. dr. J. Voorberg, molecular en cellular biologist (and Amsterdam University Medical Center)

Spaarne Gasthuis

Dr. I. van der Lee, pulmonologist

Dr. B. M. Sondermeijer, pulmonologist

St. Fransiscus Gasthuis & Vlietland Hospital

Dr. M. E. Kevenaar, internist

Drs. Y. L. Soei, internist

Dr. E. J. Wils, intensivist

St. Jansdal Hospital

Dr. F. N. Croles, haematologist

<u>Synapse Research Institute</u>

Dr. B. de Laat, biochemist, director

<u>Tergooi Hospital</u>

Prof. Dr. P. W. Kamphuisen, internist vascular medicine

Dr. R. Vink, intensivist

<u>University Medical Center Groningen</u>

Prof. dr. T. Lisman, biochemist Prof. dr. K. Meijer, haematologist, Department Haematology

Dr. Y. I. G. van Tichelaar, internist

<u>University Medical Center Utrecht</u>

Prof dr. O. L. Cremer, anesthesiologist-intensivist

Dr. G. Geersing, general practicioner, Julius Center, Department Primary Care

Dr. A. Huisman, clinical biochemist

Prof dr. H. A. H. Kaasjager, internist vascular medicine

Dr. N. Kusadasi, haematologist-intensivist

Dr. C. Maas, principal investigator coagulation & fibrinolysis

Dr. M. Nijkeuter, internist vascular medicine

Prof. dr. R.E.G. Schutgens, haematologist, Van Creveldkliniek

Dr. R. T. Urbanus, biochemist, Van Creveldkliniek

Dr. J. Westerink, internist vascular medicine

<u>Wilhelmina Hospital Assen</u>

Dr. H. J. Faber, internist-intensivist

<u>Zaans Medical Center</u>

Drs. S. C. E. Koster, anesthesiologist-intensivist

Zuyderland Hospital

Dr. P. van Montfort, resident internal medicine

Dr. D. J. L. van Twist, internist vascular medicine
